# From static definitions to dynamic landscapes: physiological states in yeast

**DOI:** 10.3389/fmicb.2026.1803305

**Published:** 2026-05-29

**Authors:** Paola Nathali Hernández-Valenciano, Alexis García-Rubio, Dania Sandoval-Nuñez, Carlos Daniel Zuñiga-Arroyo, Ernesto Borrayo, Carolina Gómez-Márquez

**Affiliations:** 1 Biodigital Innovation Lab, Department of Translational Bioengineering, Exact Sciences and Engineering University Center, Universidad de Guadalajara, Guadalajara, Mexico; 2 University of Guadalajara, Guadalajara, Mexico

**Keywords:** *K. marxianus*, physiological states, *S. cerevisiae*, Waddington landscape, yeast

## Abstract

Physiological states have traditionally been defined as discrete and static categories derived from snapshot measurements. This approach ignores the dynamic and context-dependent nature of cellular behavior. We propose a shift from static definitions to a dynamic landscape framework to describe the physiological states in yeast. Focusing on adaptation, viability, and vitality,—three states of industrial relevance,—we integrated transcriptomic analyses in *Saccharomyces cerevisiae* and *Kluyveromyces marxianus* which represent gradual transitions rather than isolated entities. Returning to Waddington's epigenetic landscape metaphor, we conceptualized physiological states as attractors in a high-dimensional space shaped by regulatory, metabolic, and environmental factors, where cell dynamics follow trajectories between attraction basins. This perspective provides a theoretical framework for integrating multi-omics data and advancing the prediction and control of cellular behavior in biotechnological systems.

## Introduction

Yeasts are an important element in targeted biological processes. The ability to monitor and control the metabolic state of these cell populations has become crucial in modern biotechnology, as it dictates the efficiency of different bioprocesses and the quality of their end products (Yook and Alper, 2025; Shi et al., 2025). While the term ***physiological state*** is widely used to describe the functional condition of a cell, its definition has evolved significantly with technological advancement, moving from simple observations and molecular characterizations to high-throughput omics technologies. For example, transcriptomics has been used to reveal the ‘transcriptomic signatures' of different states (Mulas et al., 2021; Chrysanthopoulos et al., 2010; Pandey and Zafar, 2022; Kulkarni et al., 2019).

The traditional view often treats physiological states as discrete static entities and classifies them based on snapshots of their molecular and functional properties at a given point in time. This reductionist perspective misses the biological reality of a cell's dynamic, context-dependent nature, as it evaluates traits as an isolated phenomenon. Emerging approaches have reinforced the notion that the physiological states are constantly changing over time, reflecting their continuous shifts in response to environmental fluctuations, capturing a more dynamic picture of cellular health and stress responses (Flores-Cosío et al., 2020; Hewitt and Nebe-Von-Caron, 2004).

A precise, quantitative, and integrative understanding of the integrated biological and environmental conditions that render a particular physiological state is essential for bioprocess optimization: however, a universally accepted framework remains elusive, hindering our ability to reliably predict and manipulate cellular behavior (Konstantinov, 2000). It is also clear that conceptual transitions cannot be limited to dynamic perceptions. Defining physiological states by name and boundaries is obsolete and impractical.

Although we present adaptation, viability, and vitality as physiological states, these concepts should be considered as particular additive conditions at a given time and not as independent boxes, as they are interactive cause-and-effect phenomena.

This makes a necessary conceptual transition from an approach focused on static, observable functions to a more quantitative, multidimensional view that is sensitive to cellular heterogeneity and evaluates available data from an integrated perspective. Therefore, describing a phenomenon as complex as the physiological state from a single omics dimension has proven to be insufficient, and multiple omics data should be used. Under such an integrated approach, it is possible to assemble sufficient information to infer cellular dynamism and predict cellular behavior.

To frame the concept of physiological state from multiple perspectives, we implemented a two-level approach. Yeast behavior was evaluated by two different techniques, transcriptomic analysis and transcriptome-guided metabolic contextualization. Although it is not yet a multiomic approach, it provides a proof-of-concept on how different data can be integrated in order to delineate three key functional conditions in bioprocesses: adaptation, understood as the cellular response to environmental changes; viability, as the capacity for cellular proliferation; and vitality, associated with metabolic and functional activity that guarantees efficient performance.

The transition to a multidimensional framework is not merely conceptual; it is a capability made possible by the proliferation of high-throughput omics (transcriptomics, proteomics, metabolomics, and regulomes). Currently, access to an unprecedented volume of data offers simultaneous views into different dimensions of cellular function. However, this data alone is insufficient. The concurrent development of computational systems biology, network theory, and machine learning methodologies provides the necessary tools for integrating these disparate data layers. The synthesis of massive amounts of data enables the reconstruction of high-dimensional state landscapes, rendering them computationally feasible and experimentally relevant. Although in their current state, experimental data only provide snapshots of cellular conditions, it is possible to extrapolate the information into a putative cellular dynamic flow. As real-time data recompilation improves, we will gain a better perspective of cellular dynamics.

## The physiological Waddington landscape

To move beyond this static perspective and approach physiological states as dynamic processes, Waddington's conceptualization of the epigenetic landscape provides a powerful metaphor for visualizing these processes (Waddington, 1957). The Waddington landscape has been widely used to approximate the visualization of cellular fate, transitions, and differentiation (Moris et al., 2016; Sáez et al., 2022; Sánchez-Romero and Casadesús, 2021; Camacho-Aguilar et al., 2021; Rand et al., 2021; Merkt et al., 2025). It conceptualizes the cell differentiation states as hills and valleys, where each valley corresponds to a specific cellular fate, and each slope to a possible path along which a “marble” (the cell) is directed by the “gravity” generated by the underlying regulatory networks involved in cellular differentiation (Waddington, 1957). In the pursuit of a more dynamic interpretation, physiological and cellular states, which were later described as basins of attraction in a high-dimensional landscape, are products of their gene regulatory network configuration. It has been proposed that the landscape itself is malleable, as external signals lower the barriers between attractor basins, reshaping its topology and enabling transitions between states that would otherwise remain inaccessible (Marr et al., 2016). The role of external perturbations extends beyond the reshaping of barrier heights. It has been shown that when a phenotypic demand is not met, the gene regulatory network itself undergoes an active exploratory reconfiguration until a new stable attractor satisfies the imposed demand (Schreier et al., 2017). While invaluable, the classical application of this metaphor emphasizes differentiation, a highly unidirectional process. Therefore, it is imperative to extend this concept to a more dynamic bioprocess context, in which transitions between states are continuous, reversible, and deeply influenced by external perturbations.

To translate Waddington's metaphor into a testable and quantitative model, the formalisms of dynamical systems theory are essential. In this framework, the complete cellular state at any given moment can be represented as a state vector, a point in a high-dimensional state space, where each axis corresponds to a measurable variable, such as gene expression levels or metabolite concentrations. The cellular “landscape” is mathematically defined by a set of governing rules, often derived from gene regulatory networks, that determine how this vector evolves over time. Within this space, Waddington's “valleys” can be formalized as attractors (stable configurations such as steady states or limit cycles) that correspond to the functional phenotypes a cell can adopt. A transition between states is thus not an abstract notion but a quantifiable trajectory from one basin of attraction to another, triggered by perturbations that push the cell-state vector beyond a stability threshold. Building on this dynamic perspective moving toward an integrative definition of these physiological states, they should not be considered as discrete points but as attractors within a high-dimensional state space. Each attractor is surrounded by an area (i.e.,'basin of attraction') composed of a region where different elements (cells' molecular variables, physiology, and environmental conditions) are arranged in configurations that converge on the same core functional phenotype. A transition occurs when the element's interaction “pushes” the cell out of this basin into another basin's domain and therefore changes the current cell's physiological state.

## Differential expression genes as experimental evidence

As an example of Waddington's physiological state vectors for the estimation of adaptation, viability, and vitality, we analyzed changes in gene expression. We integrated RNA-seq datasets from NCBI BioProjects for *Saccharomyces cerevisiae* and *Kluyveromyces marxianus* to identify differentially expressed genes (DEGs) at a global level across three experimentally defined physiological states. Adaptation was represented by stress-related conditions (PRJEB18911 and PRJEB25926), viability under conditions associated with cell cycle regulation and growth progression (PRJEB35742 and PRJNA658204), and vitality under optimal growth conditions (PRJEB31173 and PRJNA782397). Across both yeast models, *S. cerevisiae* and *K. marxianus*, distinct transcriptional responses were observed. In *S. cerevisiae*, the states of adaptation, viability, and vitality included 1,757, 1,287 and 5,052 DEGs respectively, whereas in *K. marxianus* 3,315 genes were differentially expressed during adaptation, 2,123 during viability, and 466 during vitality.

In addition to these state-specific responses, additional gene distributions were found to be expressed in more than one physiological state. These genes may be constitutive or represent potential transitional/overlapping functions between states, reflecting gradual molecular shifts. The overall distribution included genes exclusive to each physiological state, genes shared between the two states, potential elements involved in state transitions, and genes common to all three states, which altogether provide evidence that makes it possible to identify genes that are expressed under particular conditions and those that may correspond to basal functions essential for cellular maintenance.

Subsequently, genes uniquely expressed in each physiological state were compared across the two yeast species to identify shared transcriptional patterns. This comparison revealed that the genes were expressed in both species under analogous experimental conditions, suggesting that conserved molecular mechanisms are associated with each state. Specifically, adaptation genes, such as RFA1, MSH4, DBF2, KAR3, and SWI4, are involved in DNA damage repair processes, stress response, nuclear division, and cell cycle regulation, consistent with responses to adaptive contexts (Sampathkumar et al., 2024, 2023; Hasegawa et al., 2012; Akoto et al., 2025). For viability, BUD7 plays essential roles in the polarization of new shoots in progenitor cells, chitin biogenesis in the yeast cell wall, and intracellular transport, reflecting the key aspects associated with this state of proliferation and cell membrane maintenance (Yang et al., 2022; Trautwein et al., 2006; Cansado et al., 2021). TOP2, PHO23, CAK1, and DAD1 are involved in the key processes of transcription, chromatin organization, and cell cycle regulation, providing the cell with what is essential for maintaining vitality (Gittens et al., 2019; Loewith et al., 2000; Kim et al., 2009).

Finally, to verify the functional metabolic contextualization of each physiological state, the transcriptomic profiles were integrated into the genome-scale metabolic model Yeast9 (Zhang et al., 2024). When these data were integrated into the model, biomass flows reflected different behavioral patterns in each species, indicating diverse physiological strategies and metabolic reconfigurations for each physiological state.

The applied transcriptomic methodology based on differential expression analysis and transcriptome-guided contextualization enabled us to identify distinct signatures directly associated with each physiological state. This approach captures a static glimpse of the cellular conditions/phenomena. The progressive incorporation of additional omics layers may improve the reconstruction of physiological state transitions by naturally capturing the dynamic behavior of the cell. In this sense, the present categorization based on static gene expression profiles should be understood as an initial approach, although important gaps remain in the visualization of these transitions within the proposed framework. Even when the physiological state was addressed solely by transcriptomic data in the present work, the notion of a multidimensional/multi-parametric approach was still reinforced. It is possible to include this type of data in a multidimensional landscape, where realistic cellular models consistent with cellular behavior are constructed using all available data. From this perspective, different datasets can be included as a new dimension in the landscape in such a way that it enriches and even reshapes it. As more datasets become available, the landscape model becomes more accurate, and the prediction of the particular physiological state of the cell at a given time may be better explained.

## How it builds together

**Figure 1:**
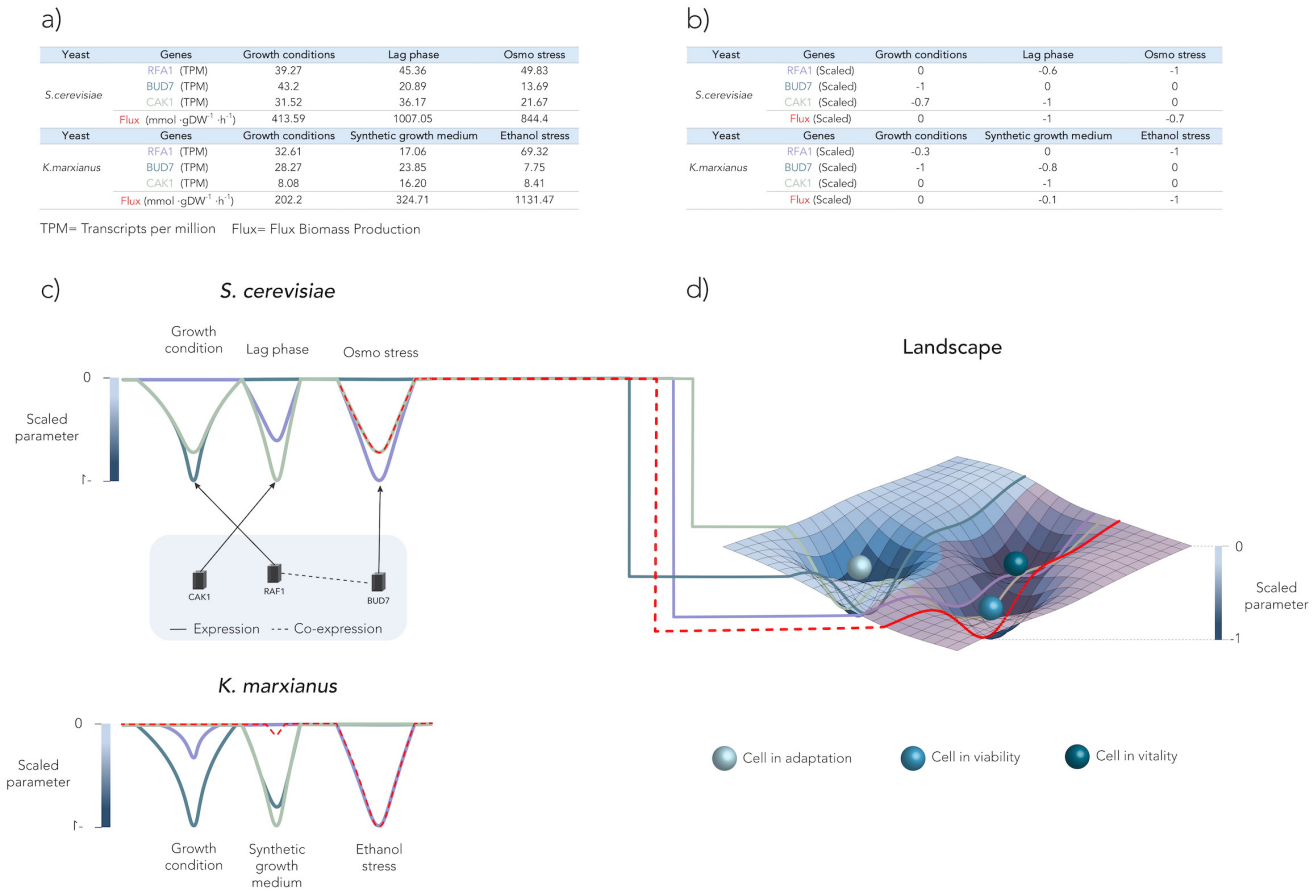
Physiological trajectories and landscapes reconstruction through integration of scaled omics data in *S. cerevisiae* and *K. marxianus*. **(a)** Raw data of absolute gene expression levels (*transcripts per million*, TPM) for genes *RFA1, BUD7*, and *CAK1*; metabolic flux data expressed as biomass production maximization (mmol gDW^−1^h^−1^). **(b)** Normalization of absolute values *z*: gene expression and metabolic flux parameters were scaled to a homogeneous range [0, −1] using the transformation $z_{i}=-\frac{x_{i}-\min\left(x\right)}{\max\left(x\right)-\min\left(x\right)}$. Under this scheme, higher expression values approach −1 while lower values approach 0. **(c)** Scaled comparative expression and co-expression representation of selected genes and metabolic fluxes across experimental conditions in *S. cerevisiae* and *K. marxianus*. **(d)** Conceptual landscape integrating traditional physiological states with data from panel c) *S. cerevisiae* and putative experimental conditions. This visualization treats cellular identity as a series of dynamic regions within a continuous, multidimensional state space.

conceptualizes cell differentiation-states as hills and valleys where each valley could be pictured as a specific cellular fate, and each slope as a possible path where a marble (the cell) is directed by the “path” generated by the different events that are involved in cellular differentiation. Similar to Waddington's landscape metaphor for cell differentiation, cellular physiological states are considered valleys in which a cell may or may not transition through different states in accordance with particular conditions. The main difference with Waddington's model is that physiological states do not behave as hills and streams that provide paths for the cell to follow, but rather as basins where the cell tends to move around. While in Waddington's perspective, the cell rolls down from shallow to deeper valleys as it further compromises differentiation, in our representation, the landscape does not have a “down” direction; hence, the cell can move in any direction across the landscape, provided the necessary conditions are met. Figure 1 shows the reconstruction of the physiological state landscape. Methodologically, this is achieved by scaling multidimensional quantitative/qualitative parameters (i.e., genetic expression, color, and metabolite concentration) that provide information about a cell's physiological condition at a particular evaluation time. The cellular response, transition, or mutation can be predicted based on its location along the reconstructed topological landscape. By establishing the conditions typically associated with a particular physiological state and the aforementioned vectors, the determined regions of the landscape can be identified as that physiological state. The current representation respects the traditional conception of two major supercategories, each containing other local minima within its main basin. As such, the physiological states that mainly impact the bioindustry are divided into these supercategories, where vitality and viability are found as the local minima of one, whereas adaptability determines the other.

As physiological states are now considered as dynamic rather than fixed or discrete categories, a given cell's physiological state describes its current state, not its transition. From this perspective, RNA velocity could provide the necessary component for mapping cellular movement among these valleys and transitions, revealing where a cell is moving, in which direction, and how quickly it will arrive at a new state (La Manno et al., 2018; Shimamura, 2025).

Therefore, physiological states act as attractors within the high-dimensional state space, and transitions between such valleys occur when cellular and/or environmental variables “push” the cell over the hills into an adjacent physiological state. As such, depending on the stimulus that a cell has in a particular environment, it will transit through this space, either falling into one of these valleys and remaining there, or continuing in accordance with the cell's own needs and metabolic capabilities. The cell is considered to transit through physiological states as the values of each trait, characteristic, and gene interaction are modified as it provides a particular response to changing environmental conditions.

Under this framework, physiological states emerge not as discrete or sequential entities; however, as dynamic configurations within a high-dimensional state space shaped by the interplay of biological regulatory variables. Landscape representation captures the inherent plasticity of cellular physiology, in which stability, fluctuation, and transition coexist as properties of the same system. According to this view, the physiological state of a cell is defined by its position within the landscape and the structure of the surrounding basins of attraction, rather than by static molecular signatures alone. This conceptualization provides a theoretical foundation for understanding physiological states as dynamic, context-dependent biological configurations, offering an integrative framework for the addition of multi-data and the interpretation of cellular behavior as a continuous process of state exploration.

Although our approach does not have a direct impact on the biotechnological use of yeast, it provides a shift in perception on how data integration may identify the environmental conditions required for a desired cellular response. This framework should be extrapolated for multiomic data integration to achieve an accurate physiological landscape.

## Data Availability

The raw data generated in this study can be found here: https://www.ncbi.nlm.nih.gov, accession numbers PRJEB18911, PRJEB25926, PRJEB35742, PRJNA658204, PRJEB31173, and PRJNA782397.
